# Monomer Selection for In Situ Polymerization Infusion Manufacture of Natural-Fiber Reinforced Thermoplastic-Matrix Marine Composites

**DOI:** 10.3390/polym12122928

**Published:** 2020-12-07

**Authors:** Yang Qin, John Summerscales, Jasper Graham-Jones, Maozhou Meng, Richard Pemberton

**Affiliations:** Faculty of Science and Engineering, School of Engineering, Computing & Mathematics, University of Plymouth, Drake Circus, Plymouth PL4 8AA, UK; J.Summerscales@plymouth.ac.uk (J.S.); jasper.graham-jones@plymouth.ac.uk (J.G.-J.); maozhou.meng@plymouth.ac.uk (M.M.); richard.pemberton@plymouth.ac.uk (R.P.)

**Keywords:** thermoplastic, natural fiber, vacuum infusion, monomer

## Abstract

Awareness of environmental issues has led to increasing interest from composite researchers in using “greener” materials to replace synthetic fiber reinforcements and petrochemical polymer matrices. Natural fiber bio-based thermoplastic composites could be an appropriate choice with advantages including reducing environmental impacts, using renewable resources and being recyclable. The choice of polymer matrix will significantly affect the cost, manufacturing process, mechanical properties and durability of the composite system. The criteria for appropriate monomers are based on the processing temperature and viscosity, polymer mechanical properties, recyclability, etc. This review considers the selection of thermoplastic monomers suitable for in situ polymerization during resin, now monomer, infusion under flexible tooling (RIFT, now MIFT), with a primary focus on marine composite applications. Given the systems currently available, methyl methacrylate (MMA) may be the most suitable monomer, especially for marine composites. MMA has low process temperatures, a long open window for infusion, and low moisture absorption. However, end-of-life recovery may be limited to matrix depolymerization. Bio-based MMA is likely to become commercially available in a few years. Polylactide (PLA) is an alternative infusible monomer, but the relatively high processing temperature may require expensive consumable materials and could compromise natural fiber properties.

## 1. Introduction

Fiber-reinforced composite materials have been widely used for marine applications (marine renewable energy devices, offshore oil/gas infrastructure, boat hulls, etc.) due to their superior resistance to biological and chemical attack in the harsh marine environment [1,2]. There is now an increasing trend to replace conventional synthetic fibers and petrochemical resins with renewable and recyclable materials for composite production [3]. Special attention to environmental issues should be taken for composites in marine applications due to the risk of microplastic contamination of the marine ecosystem [4,5]. Natural fibers (e.g., bamboo, coir, flax, hemp, jute, sisal, etc.) have attracted much interest as alternatives to conventional glass/carbon fibers in recent years [6]. There is limited literature on natural fiber-reinforced plastics (NFRP) composites in the marine environment, but the reviews by Moudood et al. [7], Al-Maharma and Al-Huniti [8] and Célino et al. [9] do consider the effects of water on NFRP. The advantages of natural fiber reinforcements include being renewable, biodegradable and low cost with high specific modulus and strength. However, the change from synthetic to natural fibers does incur a number of new considerations. There are over 100 review papers [10] addressing bast fibers and their composites published in the past 10 years, with 16 [11,12,13,14,15,16,17,18,19,20,21,22,23,24,25,26] identified with a 2020 publication date. Further, any reader seeking more information may consider our series of reviews that specifically cover fibers [27], organisms and enzymes [28], forensics [29], composites [30], modeling [31], interfaces [32], manufacture [33] and life cycle assessment (LCA) [34].

For the matrix in composites, thermoset resin composites become a problem at end-of-life, especially for large structures. Investigations into thermoplastics, as replacements for thermosets, are growing rapidly as these composites can be reprocessed, although fiber lengths are normally reduced during recycling. After pyrolysis, useable carbon fibers can be reclaimed from either matrix system, but the mechanical properties of glass fibers are severely reduced at high temperatures. Thomason [35] has suggested the glass fibers can be reprocessed to restore the properties, albeit with additional environmental burdens. Natural fiber composites at end-of-life have the possibility of incineration with energy recovery or disposal by composting with methane collection if the matrix is amenable to that option [36,37,38,39]. Bio-based or biodegradable thermoplastics are (potentially) available and could offer the opportunity for greater reduction of environmental burdens [40,41,42].

Thermoplastic composites have relatively high impact resistance [43,44,45]. Sutherland comprehensively reviewed the impact testing of marine composites [46,47,48]. In the impact testing of boat-building materials [49], thermoplastics demonstrated high specific impact strength compared with other materials due to the high level of plastic deformation. Otheguy [50] studied the impact behavior of a rigid inflatable boat manufactured with thermoplastic composites (glass fiber reinforced polypropylene) using a severe impact loading of 32 kJ (500 kg steel weight dropped from 6.5 m). The boat hull largely recovered its original shape with little chipping and no cracking at locations remote from the perforated region, which was significantly different from brittle thermosetting composites. An emergency repair method (fusion bonding repair) was successfully developed to maintain structural integrity and water tightness.

The composite manufacturing process, especially liquid composite molding (LCM), could make a significant contribution to the reduction of environmental impact. The resin transfer molding (RTM) approach uses a matched pair of mold tools to produce composite structures with high fiber volume fraction (high mechanical properties) [51,52,53,54,55,56,57]. RTM is viewed as an economical and efficient process with low styrene emission [58]. For larger structures, commercial manufacture of large structures uses (thermoset) resin infusion under flexible tooling (RIFT) [59,60,61,62,63], and the process is being adapted for (thermoplastic) monomer infusion under flexible tooling (MIFT). The hierarchy of processes is LCM > RTM > RIFT ≅ MIFT and the highest appropriate level in this list is used in the text below.

The production of thermoplastic matrix composites using the polymer in LCM is impractical due to the high melt viscosity. For LCM impregnation, in situ polymerization of a liquid monomer could be practical where the system has appropriate viscosity [64]. This review paper discusses the selection of thermoplastic monomers with the potential for producing natural fiber-reinforced marine composites via in situ polymerization infusion during MIFT. Key considerations are monomer initial viscosity, process temperature, fiber/matrix interfacial compatibility and bonding, availability of bio-sourced monomer, mechanical and thermal properties of the product, moisture uptake, environmental burdens and recyclability.

## 2. Resin Infusion under Flexible Tooling (RIFT)

**Figure 1:**
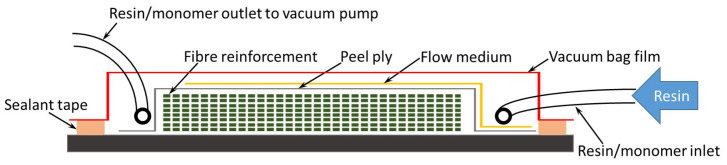
Schematic of the monomer infusion under flexible tooling (RIFT/MIFT).

RIFT [59,60,61,62,63,65] has become the process of choice for large and complex composite structures. RIFT is a set of vacuum-driven processes using one solid mold tool and a flexible counter-face tool or membrane. The basic equipment required for RIFT is just a single mold tool, a vacuum pump and consumable materials (e.g., vacuum bag, flow media, peel ply, pipework). The single solid mold face reduces the tooling cost relative to RTM. The mold clamping forces for pressure-driven RTM increase with the area, and RIFT/MIFT soon becomes the only sensible LCM option for the production of large surface area composite structures, for example, boat hulls or wind turbine blades [63,66]. RIFT II denotes the process variant where the long-range flow of the liquid precursor (the uncured polymer matrix) into a flow medium/distribution mesh floods the surface of the laminate stack before through-thickness impregnation of the reinforcement. RIFT II is known by a variety of abbreviations with Seeman Composites resin infusion molding process (SCRIMP^TM^), or vacuum-assisted resin transfer molding (VARTM) [62], dominating terminology in the United States. In this paper, RIFT is used for production with thermosetting resins, and MIFT for the in situ polymerization process to produce thermoplastic matrix composites. The layout of the RIFT/MIFT is shown in Figure 1 below.

The key factors for a successful RIFT/MIFT process are (i) polymer rheology, (ii) reinforcement permeability, which quantifies resistance to resin flow [67], and (iii) processing temperature.

### 2.1. Polymer Rheology

Rheology is the science of flow and deformation of matter. For fluids, the key characteristic is viscosity: the ability of a fluid to resist a change in the arrangement of the molecules when under an applied strain or stress [1]. Note that a low viscosity indicates a high flow rate, or a high viscosity indicates a low flow rate. The polymer industry defines two forms of viscosity:

1. Dynamic viscosity (μ): the force required to overcome internal friction. The SI units are Pascal-seconds (Pa·s: identical to 1 kg·m^−1^·s^−1^), although the composites industry often uses centimeter–gram–second (CGS) units: centipoise (cP). There is a direct numerical equivalence between millipascal-seconds and centipoise (1 mPa·s = 1 cP).
2. Kinematic viscosity (*η*): the ratio of the viscous force to the inertial force where the latter is a function of the fluid density (*ρ*). The SI units are m^2^·s^−1^, although the parameter is often given in centistokes (CGS units: 1 centistoke is 1 mm^2^/s). Hence *η* = *μ*/*ρ*.

In LCM, the process is normally modeled using Darcy’s equation [68] where, for unidirectional flow in isotropic media, the volumetric flow rate (*Q*, units: m^3^/s) of a fluid in a saturated porous medium can be expressed as:

(1) $$Q\;=\;\frac{K\cdot A\cdot \Delta P}{\mu \cdot L}$$

where *K* is a constant of proportionality known as the permeability (units: m^2^), *A* is the cross-section of the porous medium normal to the flow direction (units: m^2^), Δ*P*/*L* is the pressure gradient driving the flow (units: Pa/m) and *μ* is the dynamic fluid viscosity. For anisotropic media (composite systems), the equation should have a tensor form.

Rudd et al. [52] suggest that the most significant practical limitation on the suitability of a resin system for the LCM process is imposed by the viscosity. Resins with extremely low viscosity may be unsuitable for LCM processes as they may result in high porosity or gross voidage. Resins formulated for liquid composite molding processes typically have an initial viscosity of around 200 mPa·s. For curing thermoset resins or for in situ polymerization of thermoplastic monomers, the progress of the chemical reaction causes an increase in molecular size and a consequent increase in viscosity. At the low differential pressures used in infusion processes, the flow front becomes effectively stationary at a defined viscosity [62]. Becker [69] quotes an upper limit for viscosity in RTM of 800 mPa·s, while the non-injection point (NIP) has been defined as a viscosity of 1000 mPa·s [58].

### 2.2. Reinforcement Permeability

The permeability of the reinforcement also has a huge influence on the LCM processes. Equation (1) was originally derived for the flow of water through isotropic saturated rock to feed the drinking fountains in Dijon, France. Permeability is viewed as a unique characteristic of a saturated porous medium. The LCM process involves permeation through the multiscale pore spaces of the (an)isotropic reinforcement under two conditions: (i) unsaturated (wetting) then (ii) saturated (fully wetted).

Summerscales [70] and Park and Krawczak [71] have considered the differences in permeability for the same fabrics between the two permeation conditions. The reported difference between unsaturated and saturated permeabilities suggests that a change in surface energy occurs during wetting and dissipates some energy. Surface tension and contact angle measurements indicate that the difference in permeability arises from varying interactions at the microscopic level between the fiber and the fluid [72]. For conventional glass or carbon fibers, Kim et al. [73] and Diallo et al. [74] have reported that saturated permeabilities are always lower than unsaturated permeabilities. However, other authors have reported opposite results [67,75].

For LCM processes with natural fiber reinforcements, the permeability is more complicated. The absorption of the permeant fluid by natural fibers, and the consequent fiber swelling, lead to fiber diameter increase, which may be responsible for inconsistencies in permeability measurements for these reinforcements. Moreover, the choice of the liquid has a significant influence on the fiber-swelling ratio (wet apparent diameter/dry apparent diameter) [76,77,78,79], where “apparent” recognizes many natural fibers have non-circular cross-sections. An experimental study by Nguyen et al. [78] demonstrated that the permeability of flax fabrics showed a strong correlation to the liquid type dependent on liquid sorption into the natural fiber (swelling effect). Similar effects of liquid absorption, and consequent fiber swelling, on permeability, were also observed for jute fabrics. The average saturated permeability measured using diluted corn syrup was 23–70% smaller than when measured with motor oil [79]. Nguyen et al. [78,80] investigated the influence of liquid absorption and fiber swelling during RTM resin impregnation of flax fiber reinforcements and suggested a relationship between fiber swelling and permeability given by:

(2) $$K=\frac{{\left(1-f_{sw}^{2}\cdot V_{f}\right)}^{n+1}}{A\cdot {\left(f_{sw}^{2}\cdot V_{f}\right)}^{n}}$$

where *K* is the permeability, *f_SW_* is the fiber-swelling ratio, *V_f_* is the fiber volume fraction, and *A* and *n* are empirically derived constants. The progress of the resin flow front may be delayed when swelling of the fibers behind the flow front constraints liquid moving forwards, or may have a favorable effect by forcing resin forwards. These opposing effects should be modeled using sink and source terms in the mass conservation equation. In consequence, the permeability value in the model may need to vary with exposure time and position in the preform. Nguyen used models with varying permeability (mass source/sink terms) to obtain better agreement with the experimental flow measurements than when using a constant permeability model. When the fiber volume fraction increased, the effect from the mass sink became greater. Since the high fiber volume fraction is a major advantage of RIFT (see Section 1), different resins could lead to vastly varying effects on the fiber swelling phenomenon (permeability of the reinforcement) during the RIFT impregnation process. In consequence, the influence of monomer selection on the permeability of natural fiber reinforcements needs to be considered but is beyond the scope of this paper.

### 2.3. Processing Temperature

The in situ polymerization of the thermoplastic matrix may require elevated temperature, so the processing temperature may be a crucial parameter in infusion processes, especially for natural fiber reinforcements. The successful implementation of MIFT could be compromised by inappropriate process consumables due to high process temperatures. Table 1 summarizes the maximum working temperatures of commercially available consumables. The potential for chemical interactions within the system should also be considered (e.g., does plasticization of pipework lead to collapse under vacuum at elevated temperatures?).

Using a thermoplastic monomer with a relatively high processing temperature could cause detrimental thermal degradation of natural fibers (e.g., flax, hemp) and their composites, especially during extended process times at high temperatures [81]. Gassan and Bledzki [82] studied the thermal degradation of flax fibers and found that temperatures below 170 °C had minimal effect on fiber properties, while above 170 °C, there was a significant reduction of tenacity and degree of polymerization. Chaishome et al. [83] reported that the thermal stability of flax fibers was significantly influenced by the thermal degradation of hemicellulose and pectin: during the production of a flax fiber thermoplastic composite, the mechanical properties could be improved by (i) reducing the hemicellulose and pectin content of flax fibers, (ii) decreasing the consolidation temperature and (iii) increasing the heating rate. Moreover, Chaishome and Rattanapaskorn [81] improved the thermal stability of flax fibers by alkaline treatment to remove hemicellulose and pectin from the flax fibers. For hemp fibers, Shahzad [84] found that the thermal degradation started in the range of 150–200 °C and prominently accelerated at around 250 °C. The thermal stability of hemp fiber was also analyzed by Ouajai and Shanks [85]: thermogravimetric analysis (TGA) and differential thermal analysis (DTA) results demonstrated that untreated fiber was less thermally stable than NaOH/Na_2_SO_3_ treated fiber, with degradation starting at 205 °C and 240 °C for each, respectively.

## 3. Monomers for Infusion

Molten thermoplastic polymers normally have viscosities far in excess of those used for LCM. However, in recent years, in situ polymerization to produce thermoplastic matrix composites has grown rapidly. Van Rijswijk and Bersee [92] reviewed in situ polymerization of most thermoplastics and classified the principal systems of potential use for MIFT into:

- Ring-opening polymerization (ROP) in which cyclic molecules are opened into linear monomers or oligomers to produce high molecular weight polymers. The monomers are:
  ○ Caprolactam (e.g., DSM fiber intermediates APA-6) to produce polyamide-6 (PA6);
  ○ Laurolactam (e.g., EMS-Grivory APLC12) to produce polyamide-12 (PA12);
  ○ Cyclic butylene terephthalate (CBT) oligomers (e.g., Cyclics Corporation) to produce polybutylene terephthalate (PBT) polyester;
  ○ Cyclic bisphenol-A oligomers to produce polycarbonate;
  ○ L-lactide to produce poly(L-lactide).
- Vinyl polymerization where monomer unsaturation (double bonds) is opened to create free radicals which undergo an addition reaction to form long-chain polymers. The available monomer is:
  ○ methyl methacrylate (MMA) (e.g., Arkema Elium^®^ acrylic thermoplastic resin formulations specifically designed for RTM/MIFT manufacture of composite parts) to produce polymethyl methacrylate (PMMA).

### 3.1. Polyamides from Lactams

#### 3.1.1. Polyamide-6 from Caprolactam

Anionic polymerization of lactams is the most developed method for in situ polymerization of thermoplastics via ROP [92,93]. The ε-caprolactam monomer (C_6_H_11_NO, melt temperature, *T*_m_ = 69 °C) undergoes ROP, which is usually conducted at 130–170 °C to produce high molecular weight polyamide-6 (PA6) [94]. Conversion up to 99.3% can be achieved in 3–60 min, depending on the type and amount of activator and catalyst added [92]. Pillay et al. [95] polymerized PA6 carbon fabric composite laminates during MIFT, with an ε-caprolactam monomer of low viscosity (~5 mPa·s at 100 °C) and the final polymer matrix demonstrated conversion of ~98%. Gong et al. [96] successfully produced self-reinforced composites with PA6 fiber and PA6 matrix using RTM via anionic polymerization of ε-caprolactam. Mechanical properties of samples produced at four temperatures (140–200 °C step 20 °C) were investigated. Both tensile and flexural strength peaked when processing at 160 °C. The conversion of monomer and the void fraction of composite were all >93% and <2.5%, respectively. A comprehensive study on the vacuum infusion of PA6 composites has been undertaken [97,98,99]. Using hexamethylene diisocyanate as an activator and caprolactam magnesium bromide as a catalyst, the viscosity remained low (<100 mPa·s) for most of the injection time with a processing window (viscosity < 1000 mPa·s) up to 20 min. Final conversion was in the range of 93–97% at processing temperatures between 130 and 180 °C [97]. The infusion process was optimized by considering both polymerization temperature [98] and the choice of activator/initiator [99]. The feasibility of using vacuum infused PA6 thermoplastic composites for MW-size wind turbine blades were reported. A 10% reduction in material cost was expected relative to the epoxy counterparts [100].

Bio-based ε-caprolactam is potentially available:

- In 2014, Genomatica (San Diego, CA, USA) announced an intention to develop enzymatic pathways to produce hexamethylenediamine, adipic acid and caprolactam [101];
- In early 2018, Genomatica and Aquafil (Trento, Italy) announced a partnership to commercialize a Genomatica process for caprolactam derived from renewable feedstocks [102,103];
- Lee et al. (2019) reviewed renewable routes to obtain monomeric precursors for PA66 and PA6 from food waste [104].

#### 3.1.2. Polyamide-12 from Laurolactam

Polyamide-12 (PA12) is produced by ROP of ω-laurolactam (C_12_H_23_NO, *T*_m_ = 154 °C), with similar activators and initiators as for PA6. Unlike PA6, polymerization of PA12 must be performed above the melting temperature of the final polymer (175 °C) to avoid polymerization inhibition due to premature crystallization [105]. Therefore, the processing temperatures are usually between 180 and 240 °C and a cooling process is required to solidify the final polymer [92]. Zingraff et al. [106] investigated the RTM process for anionic polymerization of PA12 (with carbon fabrics). The initial viscosity for the molten monomer was 23 mPa·s at 180 °C and the polymerization was completed at 190 °C for 1 h. Under optimal flow conditions, the average void content of the composite plates could reduce from 15% to less than 1%. Reactive PA12 is currently marketed by EMS Chemie, Switzerland [92]. Laurolactam is normally derived from petrochemicals by butadiene trimerization to cyclododecatriene (CDT) [107]. To the best of the authors’ knowledge, no bio-based laurolactam has been identified.

#### 3.1.3. Polyamide-6/12 from Lactams

A mixture of ε-caprolactam and ω-laurolactam may be easier to process than either monomer in isolation. Rusu and Rusu [108] synthesized copolyamides with 0, 10, 30, 40 or 50% ω-laurolactam in the monomer feed with one formulation mixed at 110 °C for one minute under a nitrogen atmosphere. The mixture was then polymerized at 160 °C for 30 min. The copolymer is less likely to crystallize than the homopolymers and will consequently have reduced mechanical properties (lower density, so fewer bonds/m^3^ to react stress) and lower environmental resistance (due to greater free volume).

### 3.2. Polybutylene Terephthalate from Cyclic Butylene Terephthalate

Many researchers have reported LCM production of thermoplastic polybutylene terephthalate (PBT) composite from cyclic butylene terephthalate (CBT) oligomers. Commercial CBT was usually used in these studies, with the polymerization temperature ranging from 190–260 °C [64,109,110,111,112,113]. Commercial CBT is normally a mixture of oligomers between two and seven repeat units, i.e., from the dimer to the heptamer [111,114]. The melting point of an oligomer mixture is usually lower than the pure, discrete cyclic oligomer. CBT softens at 140 °C and melts completely at 160–190 °C, whereas the pure cyclic dimer melts at 196 °C [115] and the pure cyclic tetramer melts at 248 °C [116]. The initial melt viscosity of the oligomer mixture is 150 mPa·s at 150 °C and falls to 20–30 mPa·s at 190 °C [92,115,117], whereas the dynamic viscosity of conventional PBT was reported to be ~1000 Pa·s at 250 °C [118]. Parton and Verpoest found the final conversions of CBT oligomers were between 92 and 98% when polymerized at 190 °C for 30 min.

Pang et al. [119] and Abt and Sanchez-Soto [120] reviewed the polymerization of CBT oligomers as the precursor material for thermoplastic polyesters and their composites. The properties of the final product are highly dependent on the processing temperature. Isothermal polymerization below the *T*_m_ of PBT (∼225 °C) produces PBT with a high degree of crystallinity, which results in a brittle matrix [93,120]. Toughening of PBT may be required to improve the fracture toughness of PBT composites, e.g., by adding polycaprolactone [111,121]. CBT was a product of Cyclics Corporation (Schwarzheide, Germany). No bio-based CBT has been identified to date.

### 3.3. Polycarbonate from Cyclic Bisphenol A Oligomer

Thermoplastic polycarbonate (PC) can be polymerized from macrocyclic bisphenol A (BPA, C_15_H_16_O_2_, *T*_m_ ~200 °C) through ring-opening metathesis polymerization (ROMP). High molecular weight (*M*_w_) polycarbonate (up to 300,000) with the conversion of over 99% can be achieved by polymerizing cyclic oligomers at 300 °C for 30 min, in the presence of various basic catalysts [122].

Salem et al. successfully demonstrated that polycarbonate matrix composites (with glass fibers) could be produced by RTM [123]. At 250 °C processing temperature, molten cyclic BPA oligomer had a viscosity at ~1 Pa·s, which is comparable with the upper limit for viscosity in RTM discussed in Section 2.1. The polymerization was conducted at 300 °C to decrease the reaction time without degrading the polymer. The final BPA polycarbonate matrix showed a high polymer content of over 95% and a molecular weight of up to 50,000. Some matrix voids were observed in the composites structure, but these can be reduced by an additional consolidation step in a hot-press. To the best of the authors’ knowledge, no bio-based BPA has been found.

### 3.4. Poly(L-lactide) from L-Lactide

Polylactic acid or polylactide (PLA) is a synthetic thermoplastic polymer produced by direct condensation polymerization of lactic acid (C_3_H_6_O_3_) or by ROP of the lactide dimer (C_6_H_8_O_4_) [124]. The monomer is produced from the fermentation of 100% natural and renewable agricultural resources, such as corn [125]. The in situ polymerization of PLA based composites has attracted many studies, especially for particle-reinforced composites, due to the more homogeneous particle dispersion which could be achieved [126]. Zhuang et al. [127] reported the production of TiO_2_/PLA nanocomposite via in situ polymerization of stereoisomeric L-lactide at 150 °C for 24 h with magnetic stirring. Similarly, a graphene/PLA composite was made using the same method at 170 °C for 4 h [128].

Louisy et al. [129] reported the production of glass/poly(L-lactide) composites by RTM via in situ ROP of L-lactide with tin octoate Sn(Oct)_2_ as the catalyst. The *T*_m_ of L-lactide is ~110 °C, so to achieve low viscosity before molding, the monomer and catalyst were mixed in a 120 °C pot (maximum conversion being only 38% in this condition). The polymerization in the mold was conducted at 185 °C, and the resulting poly(L-lactide) matrices exhibited conversions and molar masses up to 99% and 78,000 g·mol^−1^, respectively.

### 3.5. Polymethyl Methacrylate from Methyl Methacrylate (Acrylic) Monomer

The methyl methacrylate (MMA, C_5_H_8_O_2_) monomer can be converted by vinyl (addition) polymerization into polymethyl methacrylate (PMMA), often simply called acrylic [92,93]. Arkema (Paris, France) Elium^®^ acrylic resin is claimed as the first thermoplastic resin compatible with RTM/infusion manufacture producing composite structures with mechanical properties similar to thermosets [130]. The viscosity and processing temperature of Elium^®^ resin are as low as 100 mPa·s and 20 °C (ambient), respectively [131]. Elium^®^ resin is widely researched for thermoplastic composites via RTM/infusion, with use increasing due to the desirable characteristics above.

Raponi et al. [132] have analyzed the thermal, rheological, and dielectric characteristics during polymerization of Elium^®^ liquid thermoplastic monomer for infusion manufacturing of composite materials. A three-step thermal cycle comprising isothermal 25 °C for 25 min, then 80 °C for 30 min, and a final isothermal 110 °C for 120 min is strongly recommended for high/full polymerization of the resin. Bhudolia et al. [133] investigated the Mode I fracture toughness of carbon fiber-Elium^®^ composites and found excellent performance (72% increase) relative to carbon fiber-epoxy composites. Obande et al. [134] compared the mechanical and thermomechanical characteristics between Elium^®^ and epoxy glass fiber composites. The acrylic composites showed equivalent modulus, 33% higher tensile strength and a 19% increase in Mode I fracture toughness against their counterparts with epoxy resin. They also proposed optimal infusion parameters to manufacture carbon fiber-Elium^®^ composites with high fiber volume fraction (up to 60%) and low void content (<1%) [135]. Some researchers have successfully produced natural fiber-Elium^®^ composites and tested their mechanical properties [136,137]. In 2016, the CANOE technical platform successfully produced a 9-m thermoplastic composite boat using MIFT of Elium^®^ at room temperature [138]. A number of commercial organizations have developed proprietary processes for manufacturing bio-based MMA. Major players in the global synthetic and bio-based MMA market include Arkema Group, Asahi-Kasei, BASF SE, Dow Chemicals, Evonik and Mitsubishi Rayon [139].

Besides the thermoplastic polymers discussed above, which have already been studied for the infusion process, some other polymers, for example, thermoplastic polyurethane (TPU), poly-(aryl) ether ketone (PEK), polyethylene terephthalate (PET) and polyphthalamide (PPA), may also be candidates for producing thermoplastic composites via infusion due to their relatively low monomer viscosities. The key parameters of the infusion process for each polymer are summarized in Table 2.

## 4. Properties of Thermoplastic Polymers

The major mechanical and thermal properties of the candidate thermoplastic polymers are summarized in Table 3 and directly influence the application of thermoplastic composites in the marine environment. As mentioned above, the viscosity and processing temperature of the monomer determine the feasibility of the infusion process and are a significant factor in monomer selection. The selection criteria are discussed in detail in Section 5 below.

## 5. Monomer Selection Criteria

The discussion above leads to a number of criteria for the selection of the monomer/resin for in situ polymerization manufacture of natural fiber thermoplastic composites for marine applications:

1. Essential criteria
  - The viscosity of the monomer must be <1000 mPa·s (NIP) to enable the infusion process.
  - The processing temperature must be <200 °C to minimize the thermal degradation of the natural fibers and to reduce the cost of consumables.
  - *T*_g_ of the cured matrix should be above the maximum use temperature to minimize the creep effect in highly stressed applications.
  - Low water sensitivity is needed to maintain proper mechanical and thermal properties in marine environments.
2. Desirable criteria
  - Monomer/resin should be bio-based or have potential bio-based sources available.
  - The open window for infusion should be relatively long to enable the production of a large-scale demonstrator/product with 3D geometry in the future.
  - The cost of the monomer/resin should be relatively low.
  - Low embodied energy and other environmental burdens of the product across the entire life cycle, and recyclable at end-of-life.

The assessment of the respective monomers is summarized in Table 4.

### 5.1. Essential Criteria

#### 5.1.1. Viscosity

As mentioned in Section 2.1, the upper limit of the viscosity for monomer infusion is 800 mPa·s; thus, TPU and PPA were not suitable as the thermoplastic matrix from the MIFT method. In addition, although the monomers of PA6 and PA12 possess extremely low viscosity, they may still be applicable by delayed infusion (in order to increase the viscosity) after adding the catalyst.

#### 5.1.2. Process Temperature

For PA12, PBT, PC, TPU, PEK, PET and PPA, it is clear that their processing temperatures are too high, which will result in thermal degradation of natural fibers. Pretreatment of natural fibers may help to maintain their properties above 180 °C [81,83,84,85], but will surely increase the environmental burdens in composite production. In addition, as discussed in Section 2.3, the high processing temperature will also increase the cost of the consumables. These factors eliminate these polymers from further consideration in the current context. The processing temperatures of PA6, PLA and PMMA are relatively low, which should lead to reduced process energy, lower cost of consumables, and no requirement for fiber pretreatment.

#### 5.1.3. Glass Transition Temperature

For applications where the polymer will be exposed to high stress, it is essential that the glass transition temperature (*T*_g_) exceeds the maximum use temperature as the material will transform from elastic/brittle to viscoelastic/tough at this temperature and become susceptible to creep deformation under sustained load. The data in Table 4 indicate that TPU and PBT do not meet the set criteria for ambient temperature use.

#### 5.1.4. Moisture Content and Depression of Mechanical and Thermal Properties

Moisture absorption is another important parameter as it has a great influence on composite properties. Davies [153] investigated the influence of seawater aging on the mechanical properties of carbon fiber-Elium^®^ composites. Elium^®^ was indicated to have a lower sensitivity to seawater than epoxy. The Elium^®^ polymer saturated in seawater at 60 °C showed a ~20% decrease in tensile strength, comparing to the virgin polymer. The fatigue performance of glass fiber-Elium^®^ composites is comparable to that of glass fiber-epoxy at similar fiber content [154]. However, there are various formulations of Elium^®^ available, and the full performance data set is not yet available. Ishak and Lim [155] studied the effect of moisture absorption on the tensile properties of pure PBT polymer and short glass fiber reinforced PBT. Results showed that at 100% RH, the tensile strength of unreinforced and reinforced PBT decreased 96% and 68%, respectively, while at 81.2% RH, the moisture influence on tensile strength was insignificant. The water absorption effect on the mechanical properties of carbon fiber reinforced PC was studied by Tanaka et al. [156]. Water was absorbed in both the PC resin and the fiber/matrix interface. For the PC resin, increasing water absorption time leads to no change in tensile modulus but a decrease in the tensile strength. For the carbon fiber/PC composite, the decrease showed only when water absorption time reached 400 h. Prabhakaran et al. [157] compared the properties of PA6 at dry and 50% RH conditions. Results demonstrated that the tensile modulus and yield strength were reduced by 65% (3.4 GPa to 1.2 GPa) and 50% (90 MPa to 45 MPa), respectively.

In the context of composites intended for marine use, the hydrophilic nature of the polymer is an important consideration. Colin and Verdu [158] observed three kinds of chemical groups:

1. non-hydrophilic groups ~generally absorb less than 0.1 w/o (weight percent) of water,
2. moderately hydrophilic groups ~generally absorb less than 3 w/o water, and
3. strongly hydrophilic groups ~saturated state generally limited to values <10 w/o water.

Although the moisture absorption of the polymer cannot directly reveal that of the composite material (other parameters, e.g., the fiber reinforcement, matrix void content and the fiber/matrix interface also have important influences), it can still provide a general idea of the rate and saturation moisture absorption of composite materials. In particular, natural fibers absorb large amounts of moisture [153], so special attention should be paid to the influence of water absorption in the composite reinforcement.

Hydrophobic polymers may not wet and bond to natural hydrophilic fibers leading to a weak fiber-matrix interface [6]. This may be a competing mechanism in the monomer/polymer selection. In a strongly hydrophilic polymer, high moisture absorption is likely to reduce the properties of the composite. In a hydrophobic polymer, moisture may weaken an already poor fiber-matrix interface, and in consequence, reduce the mechanical properties of the composite. A moderately hydrophilic polymer may be more suitable for natural fiber composites in marine environments. The achievement of optimal composite properties depends on a good fiber-to-matrix bond. This issue, and the associated coupling agents or modifiers (especially maleic anhydride polypropylene copolymer) technology, have been reviewed elsewhere for natural fiber composites [159,160,161,162].

Wright [163] plotted the fall in *T*_g_ for (thermosetting) epoxy resins (from five separate published papers) as a function of moisture content and found “as a rough rule-of-thumb” there was a drop of 20 °C for each 1% of water pick-up (data available up to 7% moisture content). Assuming similar effects of water absorption across a range of polymers, high water absorption leading to a significant reduction of *T*_g_ could be detrimental in many marine applications. For saturated PMMA at 1.92% water pick-up, the *T*_g_ was depressed by ~20 °C [164]. For PLA microspheres [165], *T*_g_ was reduced from 52 °C (~0.3% H_2_O) to 37 °C (3.5% H_2_O), implying a need for the cautious design of PLA matrix composites to be used in humid tropical environments. When exposed to solar heating, the surface temperature can be significantly above ambient temperature [166]. The response is dependent on the chemical nature of the dye/pigment, and the heating may be reduced by choosing low solar absorbance materials.

Based on the evidence above, PA6 resin is not suitable for marine composites due to the high moisture absorption (up to 11% [93]), which leads to a significant mechanical and thermal property reduction of its composites. Further, the already low *T*_g_ of PA6 falls below the ambient temperature at around 4% moisture uptake making the polymer matrix susceptible to creep in wet conditions [167].

Prabhakaran et al. [93] reported a decision-making methodology (multiple attribute decision making) in resin selection for a vacuum infused glass fiber reinforced thermoplastic wind turbine blade. They considered the viscosity, processing temperature, cost and availability of the candidate resin systems. A scoring method to evaluate, the ranking of the resin indicated: PA6 > PBT > PMMA > PPA > PA12 > PET > PC > TPU > PEK. They concluded that PA6 was the best matrix material for the wind turbine blade. Based on the additional criteria for stressed components to be used in the marine environment, PLA and PMMA are selected as the only sensible matrix materials for the matrix of natural fiber-reinforced marine composites manufactured by MIFT. It is clear that the monomer selection is significantly influenced by many aspects, e.g., the type of fiber reinforcement and the application environment.

### 5.2. Desirable Criteria

#### 5.2.1. Bio-Based Monomer

PLA monomer is produced from 100% bio-based and renewable resources (Section 3.4). Although PLA meets most of the specific requirements above, it currently requires process temperatures close to the degradation of onset temperatures for lignocellulosic materials. Furthermore, the biodegradability of PLA may lead to low durability in the marine environment [153]. A study showed that the biodegradation ratios of PLA bag and bottle packaging were ~8.4% and ~5.7%, respectively, after 365 days in the ocean water [168]. A similar degradation phenomenon of PLA in a simulated marine environment was also observed by Pelegrini et al. [169], who found the degradation of PLA composite was facilitated by natural buriti fiber reinforcement. This disadvantage of PLA resin requires further consideration.

PMMA resin does not show the drawbacks above; it almost perfectly meets the essential criteria. Among the four thermoplastic polymers discussed here, PMMA possesses the highest *T*_g_ (107 °C), minimizing the potential for creep. The cost of Elium^®^ MMA monomer is higher [93]; nevertheless, the low processing temperature could significantly reduce the cost of consumables and energy for infusion. Bio-based MMA monomers are not yet available, but they are under development as mentioned in Section 3.5, and commercial bio-based infusion MMA is likely to become available soon. Furthermore, Elium^®^ resin is weldable [131].

Soroudi and I Jakubowicz [170] have reviewed the available recycling methods, quality and costs for bio-based plastics. Álvarez-Chávez et al. [171] provided insight into the health and environmental impacts of bio-based plastics, with their analysis indicating that no commercially available or developed bio-based plastic was fully sustainable. A fundamental challenge in thermomechanical reprocessing of bio-based materials is the inherent degradation that may change the polymeric structure and hence affect the performance of the recycle. Yates and Barlow [172] reviewed LCA for bio-based polymers (focusing on PLA, PHA, and starch polymers) and found that, while reductions in non-renewable energy use (NREU) and global warming potential (GWP) can be achieved, the reported impacts from other environmental burdens were higher making it difficult to decide which materials had the lowest detriment to the environment.

An alternative disposal route for bio-based materials (natural fibers and plant-based resins) is biological waste treatment, which Hermann et al. [36] divide into aerobic composting or anaerobic digestion and ambient (≤35 °C) or elevated (50–60 °C) temperature processes. A biodegradable material is expected to reach a defined extent of degradation by biological activity under specific environmental conditions within a given time under standard test conditions [173]. Krzan et al. [174] have recently reviewed the standards and certification appropriate to environmentally degradable plastics. The biodegradation of polymeric materials under controlled composting conditions is the subject of a number of standard methods, including ASTM D5338 [175], ASTM D6400 [176], ASTM D6868 [177], EN 13432 [178] or ISO 14852 [179]. ASTM D6691 [180], ASTM D7473 [181], ASTM D7991 [182] and BS ISO 22766 [183] address test methods for measuring plastics (bio-)degradability in marine environments. However, Zumstein et al. [184] have eloquently argued that experimental observations of the carbon from degraded polymers (such as visual disappearance of plastic, plastic mass loss, a decrease in the plastic’s tensile strength, shortening of the average polymer chain length, or microbial growth) are all ill-suited to assess plastic biodegradation as the remnant material may remain a hazard until completely integrated back into the biological cycle.

Any chosen bio-based monomer should have low environmental burdens, environmental toxicology, embodied energy and CO_2_ emissions when compared to the synthetic equivalent.

#### 5.2.2. Open Window for Infusion

In the production of a PLA matrix composite by in situ polymerization during the RTM process, Louisy et al. [129] found that the working time could be up to 3 h by using L-lactide monomer. According to the technical data sheet, Elium^®^ 188 XO (a grade of Elium^®^ resin designed for natural fibers) has a processing open window of up to 60 min, a low peak exothermic temperature during polymerization. The processing open windows for both PMMA and PLA are significantly longer than the ~20 min open window of PA6 reported in [97]. The longer processing time will facilitate the production of large composite structures via resin infusion.

##### Latent Catalysts or Hardeners

Catalysts or hardeners with no activity under normal (ambient) conditions, which can be activated by an external stimulus (heat, ultraviolet, etc.) to initiate chemical reactions, are known as “latent” catalysts or hardeners, respectively. The catalysis is delayed until a single activation event triggers multiple subsequent catalytic reaction sites. The three principal initiation mechanisms are thermal-, photochemical- and mechano-catalysis. Mechano-catalysis is most likely to be used in the development of autonomous self-healing materials [185,186,187].

Subject to the identification of appropriate monomer for infusion of thermoplastic matrix composites, there could be scope for extending the open window (keeping the viscosity low until mold fill) of the system through the use of a latent catalyst or harder. Although Elium^®^ resin shows an open window up to 60 min, this could still be a significant advantage for the infusion of large-scale composite structure with 3D geometry.

#### 5.2.3. Cost

The cost of raw materials is very dependent on feedstock costs. The quantity purchased, the negotiating power of the purchaser, delivery costs, the presence of multiple competitive suppliers and the technology readiness level of the system (costs fall with scale-up of production facilities). Comparing the costs for the two remaining monomers from suppliers on Alibaba [188], L-lactide shows a higher price (€2600/ton) than MMA (€1700/ton). The cost for MMA is similar to epoxy (€1500/ton) and high compared to unsaturated polyester (€1060/ton). The additional costs of the recyclable thermoplastics may be mitigated by environmental charges imposed on thermosets at end-of-life.

#### 5.2.4. Recyclability

A main advantage of thermoplastics compared to thermosets is the potential for recyclability of thermoplastic composites as studied by many researchers [1,50,189]. The composites produced by ring-opening of monomers/oligomers (e.g., PLA) and vinyl polymerization (e.g., PMMA) should be processable by granulation and re-melting to produce short fiber composites of lower duty than the original continuous fiber-reinforced composites.

As one of the main advantages of Elium^®^ resin, Arkema offers two types of recycling processes on its website: (i) mechanical recycling and (ii) reactive recycling [190]. In mechanical recycling, the Elium^®^ composite structures are ground down to granules; these fragments can be used to make new composite parts after being heated. In reactive recycling, the granules are heat-depolymerized into initial raw monomer at a higher temperature, which can be utilized for totally new composites. Meanwhile, the remaining carbon or glass fibers can be reused.

Cousins et al. [191] comprehensively discussed four techniques for recycling glass fiber Elium^®^ composites, (i) thermal decomposition, (ii) mechanical grinding, (iii) thermoforming and (iv) dissolution. Thermal decomposition aims to decompose (and lose) the Elium^®^ resin and recycle the glass fiber reinforcement. Mechanical grinding is a mature technology in composite recycling and only requires low energy (0.29 MJ/kg composite). Ground materials made into samples by injection molding demonstrated higher tensile modulus and strength compared with virgin composites with short glass fibers. In terms of thermoforming, a curved spar cap was successfully straightened at 120 °C and 5.4 kPa for 8 h. Furthermore, a prototype skateboard was constructed with thin sheets planed from the straightened composite components. Glass fiber Elium^®^ composites can also be recycled via dissolution into their constituent parts. Although the volatile solvent was required, both fiber reinforcements and matrix materials (~90%) can be recycled for an energy cost of 4.0 MJ/kg composite. Further optimization is required to identify the most suitable recycling methods for natural fiber Elium^®^ composites.

A significant issue that could arise with a PMMA matrix is the performance when exposed to fire. At elevated temperatures, the polymer depolymerizes to produce a flammable monomer. On the night of 2 August 1973 at Douglas on the Isle of Man, Oroglas acrylic glazing was implicated in the rapid-fire spread through the Summerland leisure center. Fifty people died, and 80 were seriously injured [192].

In terms of the PLA resin composites, Le Duigou et al. [193] investigated the effect of recycling (by repeated injection molding) on the tensile properties of short flax fiber reinforced PLA composites. Results showed that the composite maintained tensile modulus even after six injection cycles. It would be interesting to investigate the feasibility of PLA fiber-PLA composites (although PLA fiber is a semi-synthetic fiber) in the marine environment, as it could offer great advantages in composite recycling.

Indicative environmental data are given in Table 5. LCA will be undertaken in the InterReg SeaBioComp project when (a) the process conditions for optimum composite production are known, (b) raw materials manufacturers have a more mature technology with enhanced efficiencies, (c) economies of scaled-up production are realized, and (d) bio-based precursors are likely to have entered the market.

## 6. Current Challenges and Future Perspectives

It is challenging to select a suitable monomer for the manufacture of natural-fiber thermoplastic composites via MIFT, as only a limited set of monomers can meet the basic criteria. PMMA does not have an available bio-based monomer yet, while PLA requires high processing temperature (high energy consumption and cost of consumables) as well as its relatively low durability. Furthermore, the biggest challenge may be a sustainable and cost-effective approach for the end-of-life thermoplastic composites dependent on the polymer matrix. Suitable systems using thermoplastics should be much higher in the recycling hierarchy than thermoset resins.

In spite of these challenges, there is an expectation that the production of natural-fiber thermoplastic composites via MIFT for marine applications (especially for large structures) is a trend that will grow rapidly, with its superior renewable and recyclable characteristics as well as low manufacturing cost. These developments will be expedited by:

- The bio-based MMA monomer being produced on at an industrial scale;
- The modification of lactide monomer resin to reduce processing temperature and enhance the durability of PLA;
- Modified, or new, monomers/polymers to meet the criteria (e.g., reduced moisture absorption of infusible polyamides) in order to introduce more candidates;
- The use of copolymer systems as the matrix.

## 7. Conclusions

The use of natural fibers as the reinforcement for “sustainable composites” calls into question the use of a thermoset resin matrix. Processing of thermoplastic matrix composites normally requires a temperature that will damage the fibers. In consequence, there is increasing interest in situ polymerization during MIFT. The monomer selection for marine applications has been discussed in this review. The parameters considered were (i) monomer viscosity, (ii) processing temperature, (iii) moisture absorption, (iv) mechanical properties, (v) bio-based availability, (vi) process open window, (vii) cost, and (viii) recyclability. It was concluded that a commercially available acrylic resin best fits the criteria for monomer selection as the thermoplastic matrix. The commercial resin is not currently bio-based but is expected to become available in the near future. Special attention should be given to the recycling of the composites to minimize the cost/energy and environmental impact; LCA is needed to confirm the sustainability. Moreover, latent catalysts may be developed to further improve the open window for the infusion. Finally, PLA resin could be an alternative, although the relatively high processing temperature and potential durability issues may limit its use.

## Figures and Tables

**Table 1:** Commercial consumables for RIFT, and their maximum working temperatures [86,87,88,89,90,91].

Consumables	Materials	Maximum Temperature (°C)
Tape	Polyamide (PA) film tape rubber adhesive	204
	Polyester tape with rubber/acrylic/silicone adhesive	177–220
	Polytetrafluoroethylene (PTFE) tape with rubber/acrylic/silicone adhesive	204, 260
	Fluoropolymer tape with rubber/silicone adhesive	204, 260
	Polytetrafluoroethylene (PTFE) coated glass fabric tape with silicone adhesive	260
	Polyimide (PI) tape with silicone adhesive	399
Bagging film	Polyethylene (PE)	48, 82
	Polypropylene (PP)	135
	Polyvinyl chloride (PVC)	121
	Polyolefin	121, 140
	Polyamide (PA) and polyolefin multilayer	155
	Polyamide (PA) + polyethylene (PE) + polyamide (PA) multilayer	170
	Polyurethane (PU)	135, 176
	Polyamide (PA) and polypropylene (PP) multilayer	180
	Polymethyl pentene (PMP) and polyamide (PA) multilayer	190
	Thermoplastic elastomer (TPE)	121–195
	Ethylene tetrafluoroethylene (ETFE) and polyamide (PA) multilayer	230
	Polyamide (PA)	120–246
	Polytetrafluoroethylene (PTFE)	315
	Polyimide (PI)	399, 426
Peel ply	Polyester	121–249
	Polyamide (PA)	160–260
	Fiberglass coated with polytetrafluoroethylene (PTFE)	288, 316
	Fiberglass coated with silicone	427
Flow medium	Low-density polyethylene (LDPE)	65
	Low-density polyethylene (LDPE) and high-density polyethylene (HDPE)	65, 100
	Polyethylene (PE)	120
	High-density polyethylene (HDPE)	93, 125
	Polypropylene (PP)	100, 150
	Polyester	170, 200
	Polyamide (PA)	177–216
Release film	Polypropylene (PP)	100
	Polyolefin and high-density polyethylene (HDPE)	120
	Polyolefin	121–157
	Polyethylene (PE)	125
	Polymethyl pentene (PMP)	193, 200
	Fluorinated ethylene propylene (FEP)	210, 260
	Ethylene tetrafluoroethylene (ETFE)	220, 260
	Fluoropolymer	260, 315
	Polyimide (PI)	405
Sealant tape	Synthetic Rubber	90–232
	Silicone	400, 427
Hose/Pipe	Polyvinyl chloride (PVC)	50
	Polyurethane (PU)	60
	Low-density polyethylene (LDPE)	90
	High-density polyethylene (HDPE)	90
	Polyethylene (PE)	90, 121
	Silicone	230, 260
Spiral	Polyethylene (PE)	80, 121
	Polypropylene (PP)	60, 120

**Table 2:** Key parameters of thermoplastic monomers for in situ polymerization infusion.

Polymer	Monomer Viscosity (mPa·s)	Processing Temperature (°C)	Bio-Based Monomer Available?	Ref.
Polyamide-6 (PA6)	~5	130–200	Yes	[94,95,96,97]
Polyamide-12 (PA12)	23	180–240	No	[92,106]
Polybutylene terephthalate (PBT)	20–150	180–260	No	[64,92,109,110,111,112,113,115,117]
Polycarbonate (PC)	1000	250–300	No	[123]
Polylactide (PLA)	-	150–185	Yes	[127,128,129]
Polymethyl methacrylate (PMMA, Elium^®^)	100	20–100	Yes *	[131]
Thermoplastic polyurethane (TPU)	800	300	No	[93]
Polyether ketone (PEK)	80	340–390	No	[92]
Polyethylene terephthalate (PET)	30	250–325	No	[140,141,142,143]
Polyphthalamide (PPA)	1000	200–290	No	[93,144]

* Should be commercially available in the near future.

**Table 3:** Overview of properties of monomer-infusible thermoplastic polymers.

Polymer	Density (g/cm^3^)	Tensile Strength (MPa)	Tensile Modulus (GPa)	Strain to Failure (%)	*T*_g_ (°C)	*T*_m_ (°C)	Moisture Absorption (%)	Ref.
PA6	1.13	85	2.0–3.8	19	40–60	219–230	6–11	[93]
PA12	1.04	50–60	1.4	300	40–50	180	1.1–1.8	[93,144]
PBT	1.31	85	1.8–2.7	30	25–60	225	0.09	[93]
PC	1.20	60	2.2	>100	150	300	0.16	[93]
PLA	1.25	70	3.6	2.4	55–65	170–200	<2	[145,146,147,148]
PMMA (Elium^®^)	1.20	66	3.17	2.6	107	-	0.5	[131,135,138]
TPU	1.20	40	0.2–2.3	>500	−8–17	140	0.1	[93,149]
PEK	1.30	115	3.7	20	228	-	0.07	[92,150]
PET	1.38	69	3	13	72	255	0.5	[93]
PPA	1.18	90	2.5–3.5	6	121–138	310–330	0.36	[93,144]

**Table 4:** The relative fit to requirements of the potential monomers for thermoplastic matrix marine composites (green is good, amber is marginal, red unlikely to be usable) [151,152]. Bio-based colors are based on Table 2; numbers are Google “results” searching for “bio-based xxx” on 14 June 2020 to indicate the level of interest.

Polymer	Essential Criteria				Desirable Criteria		Pass/Fail
	Monomer Process Viscosity (mPa·s)	Process Temperature (°C)	*T*_g_ (°C)	Moisture Absorption (%)	Bio-Based	Recyclable	
PA6	~5	130–200	40–60	6–11	✓ 196k	*T*_m_ = 219–230 °C	✕
PA12	23	180–240	40–50	<2	✓ 136k	*T*_m_ = 180 °C	✕
PBT	20–150	180–260	25–60	0.09	✓ 1110k	*T*_m_ = 225 °C	✕
PC	250–300	250–300	150	0.16	✓ 286 M	amorphous (*T*_p_ *~235 °C)	✕
PLA	-	150–185	55–65	<2	✓ 22 M	*T*_m_ = 170–200 °C	✓
PMMA (Elium^®^)	100	20–100	107	0.5	✓ 220k	depolymerize	✓
TPU	800	300	−8–17	0.1	✓ 1360k	*T*_m_ = 140 °C	✕
PEK	340–390	340–390	228	0.07	✓ 1140k	*T*_m_ = 385–413 °C	✕
PET	250–325	250–325	73	0.5	✓ 217 M	*T*_m_ = 255 °C	✕
PPA	1000	200–290	121–138	0.36	✓ 1230k	*T*_m_ = 310–330 °C	✕

* *T*_p_ represents the processing temperature.

**Table 5:** Cumulative process energy requirements (CPR), cumulative energy demand (CED) and the related cumulative CO_2_ emissions (CCO_2_) for polymeric products [194,195].

Polymer	CPR (GJ/ton)	CED (GJ/ton)	CCO_2_ (kg CO_2_/ton)
**Thermoplastics**			
PA6	90.7	122.7	4680
PC	49.3	80.3	3110
PET	33.4	59.4	2070
PLA	~	89.2	501 (2334 *)
PMA **	55.6	82.6	3740
PU	48.5	75.5	3050
**Thermosets**			
Epoxy	73.6	107.1	4680
Polyester	37.5	64.5	2390

* Total fossil global warming potential excluding CO_2_ uptake. ** PMA environmental burdens below were for “polymethyl acrylate” (may not be PMMA).
